# Role of *Plasmodium falciparum *thrombospondin-related anonymous protein in host-cell interactions

**DOI:** 10.1186/1475-2875-7-63

**Published:** 2008-04-22

**Authors:** Reetesh Raj Akhouri, Ashwani Sharma, Pawan Malhotra, Amit Sharma

**Affiliations:** 1 Structural and Computational Biology Group, International Centre for Genetic Engineering and Biotechnology, New Delhi, India; 2 Malaria Research Group, International Centre for Genetic Engineering and Biotechnology, New Delhi, India

## Abstract

**Background:**

Thrombospondin-related anonymous protein (TRAP) is essential for sporozoite motility and for liver cell invasion. TRAP is a type 1 membrane protein that possesses multiple adhesive domains in its extracellular region.

**Methods:**

*Plasmodium falciparum *TRAP (PfTRAP) and its subdomains were expressed in a mammalian expression system, and eleven different mutants generated to study interaction of PfTRAP with liver cells. Binding studies between HepG2 cell extracts and PfTRAP were performed using co-immunoprecipitation protocols.

**Results:**

Five different amino acid residues of PfTRAP that are involved in liver cell binding have been identified. These PfTRAP mutants bound to heparin like the wild type PfTRAP thereby suggesting a non-heparin mediated binding of PfTRAP to liver cells. Three Src family proteins -Lyn, Lck and CrkL which interact with PfTRAP are also identified. Liver cell extracts and immunoprecipitated Src family kinases phosphorylated PfTRAP at multiple sites. An analysis of multiple TRAP sequences revealed Src homology 3 domain (SH3) binding motifs.

**Conclusion:**

Binding of PfTRAP to SH3-domain containing proteins like Src-family kinases and their ability to phosphorylate PfTRAP suggests a novel role for PfTRAP in cell signaling during sporozoite invasion and homing inside the liver cells. These data shed new light on TRAP-liver cell interactions.

## Background

Malaria is a major parasitic disease that claims more than two million lives and causes more than 500 million clinical cases every year [1]. Despite continuous efforts to control malaria, it remains a major health problem in the tropical world, mainly due to *Plasmodium falciparum*. Since the search for a malaria vaccine remains elusive, there is a constant and pressing need to identify new drug and vaccine targets. Malaria infection in humans starts when infected female *Anopheles *mosquitoes take their blood meal and inject sporozoites into the host (human) skin. Although, it remains unclear how sporozoites reach hepatocytes, experimental evidence suggests role of various molecules for successful infection that includes components both from the host and the parasite. Apicomplexans lack a classical locomotory organelles like flagellum or cilium but show an actin based locomotion called gliding motility [2,3]. Secretory organelles, including rhoptries and dense granules, are present in apicomplexans which secrete a number of proteins required for infectivity, motility and invasion [4]. Two important surface molecules that play key roles in liver cell infection are the circumsporozoite protein (CS) and the thrombospondin-related anonymous protein (TRAP). CS is a multifunctional protein that is involved in sporozoite development in mosquitoes, invasion into mosquito salivary gland and into human liver cells [5-11]. TRAP is stored in the micronemes [12] and becomes surface exposed at the sporozoite anterior tip when parasite comes in contact with host cells [13]. TRAP also plays an important role in liver cell invasion of sporozoites by helping sporozoites in gliding motility and in recognition of host receptors on the mosquito salivary gland and hepatocytes [14-18].

The *P. falciparum *TRAP (PfTRAP) extracellular domain (ECD) consists of three domains/motifs that include the A-domain (similar to the A-or I-domain which is found in integrins), the TSP (thrombospondin repeat motif, a heparin-binding module, also called the RII region) and a proline-rich segment at the C-terminus. Sequence analysis of the proline-rich segment revealed the presence of SH3-domain binding PxxP motifs in *Plasmodium *TRAPs. To understand the role(s) for multiple adhesive domains of PfTRAP, its extracellular domain (ECD) and subdomains were expressed using a mammalian expression system. Five crucial amino acid residues -C55, D162, C205, R307 (of RGD) and S416, that are likely to be involved in TRAP-liver cell interactions, were mapped. Surprisingly, most of these mutants did not show reduction in heparin-binding abilities, suggesting that the significant reduction in TRAP mutants binding to HepG2 cells was not due to loss of heparin binding ability. Interaction of PfTRAP with host SH3-domain containing tyrosine kinases -CrkL, Lck and Lyn that belong to the c-Src kinase family was also studied. Immunoprecipitation experiments indicated that each of these three tyrosine kinases and total HepG2 cell extract were able to phosphorylate PfTRAP at multiple sites. Together, these data indicates non-heparin based interaction of PfTRAP to liver cells and also suggests a role for PfTRAP in signaling during sporozoite invasion of hepatocytes.

## Methods

### Cloning of PfTRAP and its subdomains in mammalian expression vector pHL

PfTRAP A-domain, A+RII, A+RII+RGD and ECD were PCR amplified using forward primer 5'GCACATACCGGTAGAGATGTGCAAAACAATATAGTG 3' and reverse primers 5'GCACCCTCGAGACTATCCACTACCACTACCACTACCTTTGCTGTCCATGCAGAATCAGCATAC 3' (for A domain), 5'GCACCCTCGAGACTATCC ACTACCACTACCACTACCTTAACATCTAATGGTTCCCATTTTGG3' (forA+RII), 5'GCACCCTCGAGACTATCCACTACCACTACCACTACCTTTGGTTTTTGGACAGAAGAATTATC3' (forA+RII+RGD) and 5'GCACCCTCGAGACTATCCACTACCACTACCACTACCTTTTTATATTTATTATCTGATTCTCCTTTTTT 3' (for ECD). The amplified PCR fragments were digested with *Age*I and *Xho*I and fragments were cloned in pHL vector [19]. Recombinant clones were identified by restriction digestion of plasmid DNA obtained from colonies after transformation of the ligation mix. The clones were further confirmed by automated DNA sequencing.

### Expression analysis of PfTRAP subdomains in HEK 293 cells

Three clones each of PfTRAP and its fragments were selected. DNA (2 μg) of each of these were added to 100 μl of incomplete DMEM. PEI (6 μg) was then added to each mix and incubated at room temperature for 15 min. In parallel, HEK 293 cells at 60–70 percent confluency (in 35-mm tissue culture plates) were washed with PBS to remove serum. Cells in the incomplete DMEM were incubated with transfection mix for 3–4 h at 37°C in a humidized CO_2 _incubator, and 2 ml of expression media (iDMEM pH7.2+2%FBS+1× non-essential amino acid+ 1× antibiotic and antimycotic+2 mM L-glutamine) were added to each well. The media that contained secreted protein were harvested four days post-transfection and cleared by centrifugation at 5,000 rpm. Expression of recombinant proteins was detected using western blotting with anti-penta-his and anti-PfTRAP antibodies.

### Purification of PfTRAP subdomains from HEK-293 cells

Large-scale transfections (~4 litres) were carried out for pHL-PfTRAP constructs. The media were harvested from each plate and centrifuged at 5,000 rpm for 20 min at 4°C. Supernatants were filtered through 0.2 μ-filter membrane and concentrated in a diafiltration unit (Millipore) containing filtration cartridge of 10 kDa cut off. The media were dialyzed against PBS supplemented with 500 mM NaCl (pH 8.0) to remove chelators from the media. The harvested media containing PfTRAP proteins were passed through chelating column (Amersham Biosciences) that had been charged with Ni^2+ ^and pre-equilibrated with the dialysis buffer (PBS supplemented with 500 mM NaCl pH 8.0). Columns were washed with 10 column volumes of dialysis buffer, and then with 30 and 50 mM imidazole buffer. The bound proteins were eluted in 200 mM imidazole elution buffer. Eluted protein fractions were further concentrated in a 10 kDa centriprep (vivaspin) and subjected to gel exclusion chromatography (GPC). Purity of the proteins was assessed on 12% SDS-PAGE. Similar protocols were used for PfTRAP subdomains and mutants.

### Binding of PfTRAP ECD and subdomains to HepG2 cells

1 × 10^5^HepG2 cells were seeded in each well in a 96 well cell culture plate and were allowed to grow for 24 h. Media were discarded and cells were fixed with 4% (w/v) paraformaldehyde in PBS for 10 min. Fixed cells were washed and blocked with 2% BSA for two hours. Cells were incubated with equimolar (250 nmoles) amounts of PfTRAP ECD and its subdomains for one hour. Cells were washed and incubated with PfTRAP ECD antibody raised in rabbit followed by incubation with HRP conjugated anti-rabbit antibody. Binding was detected at 490 nm using OPD as substrate.

### Binding assay of PfTRAP ECD with hepatocytes (HepG2 cells and Huh-7 cells) and kidney cells (HEK 293 cells)

Hepatoma cells (HepG2 cells, Huh-7 cells) and HEK 293 cells (kidney cell line) at a confluency of 60–70% were dislodged using an enzyme free dissociation buffer (Gibco Life Sciences). Cells were washed in PBS and viability assay was performed to check for the percentage cell viability. FACS analysis was performed with hepatocytes and kidney cells HEK 293 cells. From 90–95% viable cell population, 1 × 10^6 ^cells were added to each microfuge tubes and washed thrice in FACS buffer and suspended in 50 μl of FACS buffer (PBS, pH 7.4). Cells were then incubated in FACS buffer supplemented with 1% BSA and incubated on ice for one hour. Biotinylated PfTRAP ECD protein (prepared using Pierce reagent Sulfo-NHS LC-LC Biotin) was added in equimolar amounts to each tube and allowed to bind with cells for one hour on ice. The cells were washed three times with FACS buffer, and streptavidin-PE (Calbiochem) was added to each tube at a dilution of 1: 2000. It was allowed to incubate for one hour on ice in dark. The unbound dye was washed with FACS buffer and stained cells were fixed in PBS containing 0.1% paraformaldehyde. The number of stained cells were counted using FACS and the mean value of stained cells was obtained using FACS analysis software. Stained cells alone and biotinylated-BSA bound to cells were used as controls in these experiments.

### Site-directed mutagenesis of PfTRAP ECD

Single site mutants of PfTRAP ECD were constructed using the Quick-change site directed mutagenesis kit from Stratagene, USA. The mutants were generated using primers shown in Table 1 and Table 2. Mutations were confirmed by automated DNA sequencing. The expression profile of the mutants was analyzed using HEK 293 cell transfection and western blot analysis of post-transfection media in the mammalian system.

**Table 1:** Sequence of oligos used for site directed mutagenesis of PfTRAP

P18	gTTgTTATATTAACAgCTggAATTCCAgATAgT
P19	ACTATCTggAATTCCAgCTgTTAATATAACAAC
P20	AgATTTCTTgTAggTggTCATCCATCAgATggT
P21	ACCATCTgATggATgACCACCTACAAgAAATCT
P22	CCATCAgATggTAAAggTAACTTgTATgCTgAT
P23	ATCAgCATACAAgTTACCTTTACCTTTACCATCTgATgg
P24	gTTTgggACgAATgggCTCCATgTAgTgTAACT
P25	AgTTACACTACATggAgCCCATTCgTCCCAAAC
P26	TgTggAgAAgAAAgAgg TCCTCCAAAATgggAA
P27	TTCCCATTTTggAggACCTCTTTCTTCTCCACA
P28	gATCAACCTAgACCAggAggAgATAATTCTTCT
P29	gACAgAAgAATTATCTCCTCCTggTCTAggTTg
P30	AgAAATATTCCATATgCACCATTACCTCCAAAA
P31	TTTTggAggTAATggTgCATATggAATTCT
P32	AgAAAATATAACgATgCTCCAAAACATCCTgAAAgg
P33	CCTTTCAggATgTTTTggAgCATCgTTATATTTTCT

**Table 2:** Oligos used in the sets of forward and reverse primers to generate mutations in the PfTRAP polypeptide.

Forward Primer	Reverse Primer	Residue mutated
P12	P13	C42G
P14	P15	C55G
P16	P17	T131A
P18	P19	D162A
P20	P21	C205G
P22	P23	C212G
P24	P25	S251A
P26	P27	S285G
P28	P29	R307G
P30	P31	S416A
P32	P33	T470A

### HepG2 cell binding of PfTRAP ECD and its mutants

HepG2 cells were cultured in complete DMEM at 37°C in a humidified CO_2 _incubator. 1 × 10^5 ^cells were seeded in a 96-well cell-culture plate and were allowed to grow for 24 h. HepG2 cells were fixed with 4% (w/v) paraformaldehyde for 10 min at room temperature and blocked with 2% BSA for two hours at room temperature. Equal amounts of PfTRAP ECD and its mutants were incubated with HepG2 cells for one hour and bound proteins were quantified using anti-PfTRAP antibodies. Wild type PfTRAP ECD was used as positive and quantitative control for the binding of mutant proteins to HepG2 cells.

### Preparation of HepG2 and HEK 293 extracts

HepG2 cells and HEK 293 cells were scraped from the monolayer culture flasks using enzyme free cell dissociation buffer (Gibco). Cells were washed with PBS to get rid of any other contaminant elements. Washed cells were lysed using Down's homogenizer and the cell extracts were solubilized in 250 mM (final concentration) n-octyl-beta-D-glucopyranoside buffer. The extracts were further ultracentrifuged at 100,000 g using 60Ti (Beckmann) rotor to remove insoluble matter.

### Phosphorylation of PfTRAP and its fragments by HepG2 cell extracts

PfTRAP ECD, the A-domain and the A+RII domains (2–5 μg) were incubated with HepG2 or HEK 293 whole cell extracts in the presence of ATP at 37°C for 30 min. These PfTRAP fragments were immunoprecipitated using polyclonal anti-PfTRAP antibodies that had been pre-adsorbed on protein A Sepharose beads. Beads were washed in PBS supplemented with 50 mM n-octyl-β-D-glucopyranoside and 500 mM NaCl. The sample was again washed in PBS thrice, boiled and resolved by SDS-PAGE. PfTRAP protein bands were cut and sent for LC-MS/MS analysis. Sample containing [γ-^32^P] ATP in gels were dried and phosphorylation was detected by autoradiography. HepG2 cells extract alone was taken as a negative control and PfTRAP alone with ATP was taken as a nonspecific and autophosphorylation control.

### Detection of PfTRAP phosphorylation using phosphospecific antibodies

PfTRAP ECD protein was incubated with whole cell extract of HepG2 and HEK 293 cells and in presence of cold ATP at 37°C for 30 min. The reaction mixture was immunoprecipitated using anti-PfTRAP antibody pre-adsorbed on protein A-sepharose beads. Samples were boiled and resolved on SDS-PAGE. Phosphorylation of PfTRAP was detected using phosphospecific antibodies against serine, threonine and tyrosine residues. Negative controls of HepG2 cells extract (with ATP without PfTRAP) and PfTRAP (with ATP without HepG2 cell extract) were used in these experiments.

### Binding of PfTRAP to tyrosine kinases

HepG2 cell membrane extracts were incubated with 5 μg of purified PfTRAP ECD for 30 min at 4°C in the presence of protease inhibitor cocktail and ATP. To determine the molecule(s) interacting with PfTRAP ECD, the mixture was immunoprecipitated using anti-PfTRAP antibody pre-adsorbed on beads. The beads were then washed in PBS supplemented with 50 mM n-octyl-β-D-glucopyranoside and 500 mM NaCl, and samples were boiled, resolved on 12% SDS-PAGE. Presence or absence of tyrosine kinases was detected using specific antibodies against c-Src, Grb2, Lyn and Lck and CrkL. Similar experiments with cytoplasmic and membrane extracts were performed to detect any serine and threonine kinase interactors.

### Phosphorylation of PfTRAP with tyrosine kinases proteins

Antibodies coupled to protein A beads were used to pull down CrkL, Lck, and Lyn from HepG2 membrane extracts. The PfTRAP ECD protein (20 μg) was incubated with above beads in the presence of [γ-^32^P] ATP at 37°C for 1 hr. PfTRAP was eluted from the mixture using 1M NaCl, immunoprecipitated using anti-PfTRAP antibodies, resolved on SDS-PAGE and detected by autoradiography after two days of exposure.

## Results

### PfTRAP binding to hepatocytes

**Figure 1:**
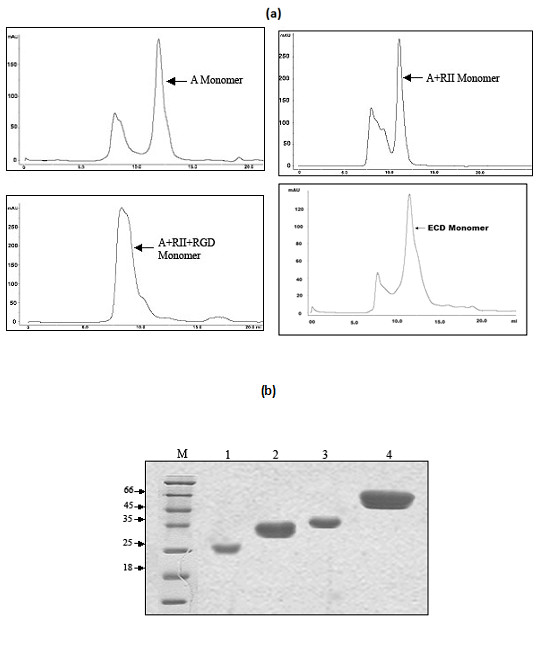
**(a). **Gel-filtration analysis of recombinant TRAP ECD and subdomains. Ni-affinity-purified recombinant PfTRAP A domain, A+RII domain, A+RII+RGD domain and ECD expressed in HEK 293 cell migrate on a gel-filtration columns (GPC) as monomers. **(b). **SDS-PAGE of TRAP subdomains under reducing conditions. The mammalian cell-expressed and purified PfTRAP A domain (lane 1), A+RII domain (lane 2), A+RII+RGD domain (lane 3) and ECD (lane 4) migrate at a molecular mass of approx. 33, 36, 38 and 70 kDa under reducing conditions.

**Figure 2:**
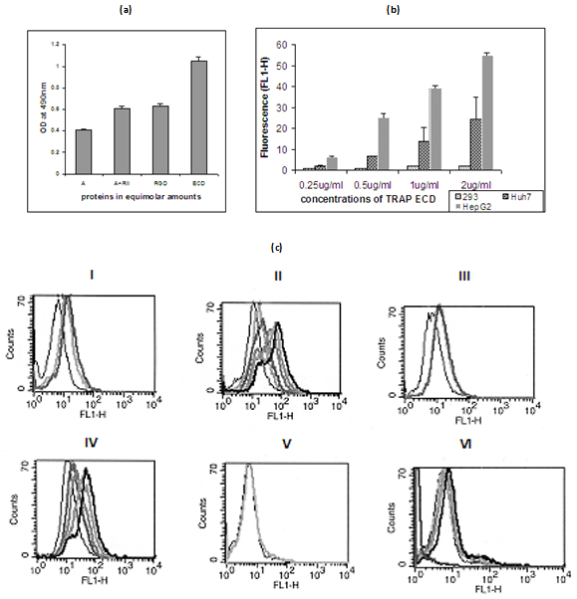
**(a). **Binding of TRAP A domain, A+RII domain and ECD to HepG2 cells. HepG2 cells (approx. 1 × 10^5^) cells were seeded in 96 well cell culture plates and incubated with PfTRAP fragments in equimolar amounts. Binding was evaluated through ELISA. Results are the mean ± SD of n = 3 experiments. **(b). **Binding specificity of PfTRAP ECD with hepatoma cell lines. HEK 293 (Kidney cell line), Huh-7 and HepG2 (hepatoma cell lines) cells were incubated with biotinylated PfTRAP ECD. Binding was evaluated through a flow cytometry analysis using Streptavidin-PE. Results are the mean ± SD of n = 3. **(c) **(i, iii and v) Control FACS data with unstained (black), stained (light grey) and Biotinylated BSA (dark grey) using HepG2 cells, Huh-7 cells and HEK 293 kidney cell lines respectively. (ii, iv and vi) FACS data to evaluate binding of PfTRAP ECD to HepG2 cells, Huh-7 cells and HEK 293 cell lines respectively with the increasing amounts of PfTRAP ECD (grey to thick black) (0.25, 0.5, 1.0 and 2.0 μg) and the stained cells control (thin black).

PfTRAP ECD and its subdomains were expressed in mammalian expression system and recombinant proteins were purified from media using Ni-ion chelating affinity chromatography and gel filtration chromatography. As shown in Figure 1a, recombinant PfTRAP protein and its fragments migrated as monomers and were of very high purity (Figure 1b). The recombinant proteins (ECD, the A-domain and the A+RII domains) were biotinylated and were assessed for their ability to bind to HepG2 cells by ELISA and FACS based assays. As shown in Figure 2a, these proteins bound to HepG2 cells with differential degree of binding affinity. PfTRAP ECD showed highest level of binding in comparison to other fragments. This binding was specific to liver cells as PfTRAP ECD bound to hepatocytes in a dose dependent manner but it failed to bind kidney cell line HEK 293 cells (Figures 2b and 2c).

### Asp162, Cys205 and Arg307 are important for PfTRAP-HepG2 binding

**Figure 3:**
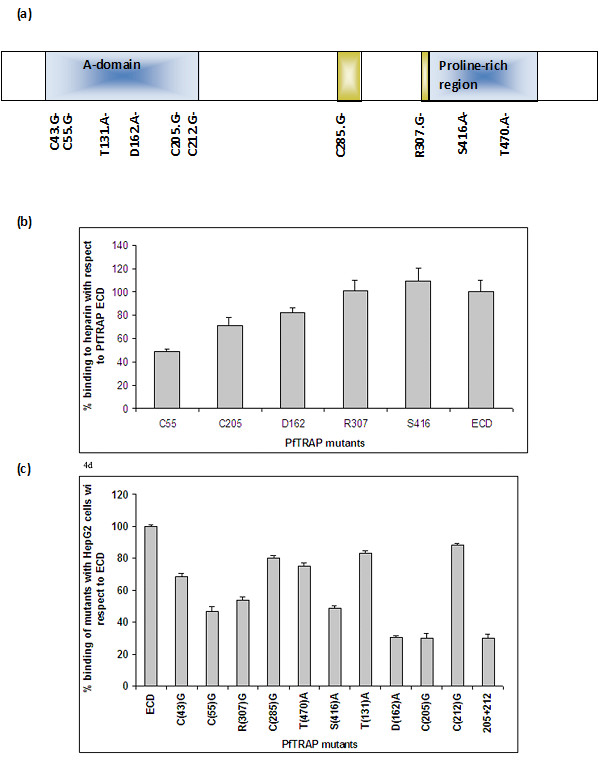
**(a) **Graphical presentation of PfTRAP mutants. **(b) **Binding of purified recombinant TRAP ECD and its mutants to heparin (100 μg) coated on ELISA plates. They were incubated with equimolar amounts of mutants. Binding was evaluated in an ELISA based assay using PfTRAP antibodies. Results are the mean SD of n = 3 experiments. **(c) **Binding of the purified recombinant TRAP ECD and its mutants to HepG2 cells. HepG2 cells (approx. 10^5^) cells were incubated with ECD and its mutants in equimolar amounts. Binding was evaluated in an ELISA based assay using PfTRAP antibodies. Results are the mean ± SD of n = 6 experiments.

To identify amino acid residues involved in interaction of PfTRAP to HepG2 cells, different PfTRAP mutants were constructed (Figure 3a) and the mutant proteins were purified to homogeneity by protocols described for the wild type protein (data not shown). These mutants were generated based on modeled structure of PfTRAP [20]. The modeling data suggested that Cys205 and Cys 212 were likely to be exposed on the surface of PfTRAP and Cys 43 and Cys 235 were likely to be linked by a disulfide bond.

The modeling suggested that T131 may be in an exposed loop, while D162 may be part of MIDAS motif. Mutants of T131 and D162 have been previously shown to affect sporozoite infectivity to salivary gland and liver cells [14]. A mutant R307 was also generated as it is part of RGD motif that has been shown to be involved in integrin interactions [21,22]. Purified mutants and wild type ECD were incubated with hepatocytes and their binding ability was assessed by ELISA assays described previously [20]. As shown in Figure 3c, two mutants C205-A and D162-A showed ~71 and ~70% reduced binding to HepG2 cells in comparison to wild type PfTRAP ECD. Mutants C43 and C55 (in A-domain) showed reduced binding by up to ~32% and 54% respectively. Intriguingly, arginine 307, which is part of conserved RGD motif and for which no specific biological significance in PfTRAP has so far been documented, appeared important for TRAP binding to HepG2 cells. Mutation of R307, which remains conserved even in field isolates of *P. falciparum *TRAP, showed ~47% reduction in binding to HepG2 cells (Figure 3c). Surprisingly, most of the TRAP mutants except C55 bound to heparin in a similar fashion as the wild type PfTRAP (Figure 3b). TRAP mutant C55 showed about 50% reduction in heparin binding. These results suggest a potential role for these five residues in non-heparin mediated binding to HepG2 cells.

### PfTRAP undergoes phosphorylation in presence of hepatocyte extract

**Figure 4:**
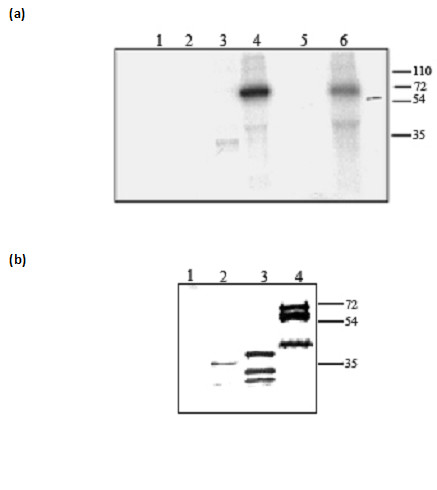
Recombinant PfTRAP undergoes phosphorylation in the presence of HepG2 and HEK 293-cell lysates. Recombinant PfTRAP fragments were incubated with HepG2 cell extract **(a) **and HEK 293 cell lysate **(b) **in the presence of P^32 ^[γ ATP]. PfTRAP fragments were immunoprecipitated using anti-PfTRAP antibody and phosphorylation status was checked through autoradiography. **(a) **Lanes 1, HepG2 cell lysate; 2, A domain+HepG2 cell lysate; 3, A+RII+HepG2 cell lysate; 4, ECD+HepG2 cell lysate. ECD phosphorylation was also checked using recombinant MAPK (lane 6). MAPK was used as a control (lane 5). **(b) **Lanes1, HEK 293 cell lysate; 2, A+RII domain+ 293 cell lysate; 3, A+RII+RGD+ 293 cell lysate; 4, ECD+ 293 cell lysate.

**Figure 5:**
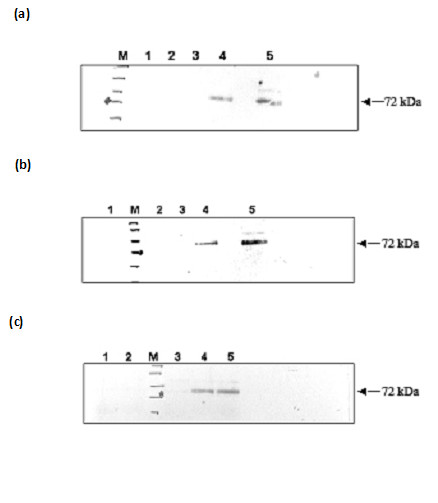
Recombinant PfTRAP ECD is phosphorylated at multiple residues. PfTRAP ECD was either incubated with HepG2 cell or HEK 293 cell lysate and immunoprecipitated using PfTRAP antibodies. Phosphorylation status was analyzed by western blotting using monoclonal antibodies against phosphorylated serine threonine and tyrosine (a, b and c). Lanes 4 and 5 show the phosphorylated PfTRAP ECD in presence of HEK 293 and HepG2 cell lysate respectively. HepG2 lysate alone (lane 1, panels a, b and c), HEK293 lysate alone (lanes 2 of a, b and c) were taken as controls. Autophosphorylation of ECD was also analyzed (lane 3, panels a, b and c) and none was observed.

To identify host molecules interacting with PfTRAP ECD, we prepared membrane extracts of HepG2 cells in a mild neutral detergent n-Octyl-β-D-glucopyranoside (USB chemicals) and incubated it with PfTRAP ECD. The reaction mixture was immunoprecipitated with anti-TRAP antibodies and TRAP band was subjected to mass spectrometric analysis. These results indicated phosphorylation of PfTRAP at multiple sites (data not shown). Autophosphorylation of PfTRAP was ruled out as no specific binding of ATP or autophosphorylation of PfTRAP was observed in the presence of [γ-^32 ^P] ATP without the HepG2 cell extract (Figure 4a). Similar phosphorylation results were observed when HEK 293 cell lysate was used in place of HepG2 cell lysate (Figure 4b). To identify the domains of PfTRAP that undergo phosphorylation, binding reactions were performed with PfTRAP subdomains in presence of [γ-^32 ^P] ATP and HepG2 cell lysate. The results showed that both PfTRAP ECD and PfTRAP A+RII proteins were phosphorylated while PfTRAP A-domain was not phosphorylated. To map sites that undergo phosphorylation in PfTRAP, the reaction mixture containing PfTRAP and HepG2 cell extract was immunoprecipitated with anti-PfTRAP antibodies and the precipitated samples were analysed by immunoblot analysis using phosphospecific antibodies. As shown in Figure 5, multiple phosphorylation sites were observed for PfTRAP-serine, threonine and tyrosine sites (Figure 5). A phosphorylation reaction of PfTRAP was carried out in presence of MAPK. As shown in Figure 5, PfTRAP got phosphorylated at multiple sites as previously seen with HepG2 cell extract. Together these results suggested that PfTRAP probably undergoes phosphorylation when released from the sporozoites at the time of HepG2 cell invasion and kinases in the HepG2 lysate phosphorylate the PfTRAP.

### Tyrosine kinases from hepatocytes bind and mediate phosphorylation of PfTRAP

**Figure 6:**
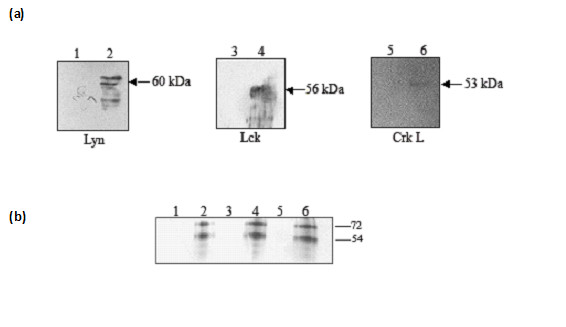
**(a) **Recombinant PfTRAP interacts with tyrosine kinase proteins. HepG2 cell membrane extract were incubated with PfTRAP ECD. The complex was immunoprecipitated using PfTRAP antibodies, followed by western blot analysis using monoclonal antibodies against Lyn (i) (lane 2), Lck (ii) (lane 4), CrkL (iii) (lane 6). To check nonspecific interaction, HepG2 cell membrane extract alone was immunoprecipitated using PfTRAP antibodies (Lane 1, 3 and 5). **(b) **Recombinant PfTRAP is phosphorylated in the presence of CrkL, Lck and Lyn proteins. Lck (lane 2), Lyn (lane 4) and CrkL (lane 6) proteins were immunoprecipitated from HepG2 membrane extract and PfTRAP was incubated with the complex in the presence of γ33 ATP. PfTRAP was immunoprecipitated from the mixture and the phosphorylation was analyzed using autoradiography.

To identify the kinase(s) in HepG2 cell extracts that are able to phosphorylate PfTRAP, immunoprecipitations of reaction mixtures were carried out with anti-PfTRAP antibodies. The precipitated samples were analysed for presence of Lck, CrkL Lyn, c-Src and Grb2 proteins in an immunoblot assay using kinase specific antibodies. As shown in Figure 6a, specific interactions of PfTRAP with Lck, CrkL and Lyn proteins were observed, while no such interaction could be seen with the c-Src and Grb2 proteins. Since Lck, CrkL and Lyn proteins also carry kinase domains, the phosphorylation status of PfTRAP was analysed in presence of Lck, CrkL and Lyn proteins isolated from HepG2 cell lysate. Phosphorylation reactions showed that PfTRAP was phosphorylated when incubated with immunoprecipated Lck, CrkL and Lyn proteins, while γ-^32^ATP alone did not phosphorylate TRAP (Figure 6b). Together, these results indicated that PfTRAP bound to and is phosphorylated by src-family kinases.

## Discussion

PfTRAP has been previously suggested to bind to HepG2 cells by interacting with sulfated proteoglycans. These studies were carried out with multimeric or soluble aggregates of recombinant PfTRAP [15,16,23,24]. In the present study, recombinant PfTRAP and its subdomains were produced in monomeric, properly folded forms using a mammalian expression system. The expressed molecules were secreted out of the mammalian cells due to the presence of signal sequence at the N-terminus of the expressed proteins. Eleven different mutant TRAP proteins were generated to map the amino acid residues involved in PfTRAP binding to HepG2 cells. Results of HepG2 cell binding assays with mammalian expressed PfTRAP and its subdomains showed differential binding abilities. PfTRAP ECD bound to HepG2 cells more strongly than its other two fragments (PfTRAP A+RII and PfTRAP A). The HepG2 cell binding of TRAP was specific as neither PfTRAP ECD nor its two recombinant subdomains were able to bind HEK 293 cells (kidney cells). These results were in agreement with a previous study where a baculo-expressed PfTRAP A domain was expressed in monomeric forms [20].

Amino acids involved in binding of PfTRAP proteins to HepG2 cells were evaluated. Five mutants (D162, C205, C55, R307 and S416) exhibited 40–70% reduced binding to HepG2 cells. Specifically, the reduced binding of R307 (a part of the RGD triplet) indicates that this conserved sequence plays an important role in binding of PfTRAP to HepG2 cells via yet unidentified receptor(s) on liver cells. Surprisingly, the-T131A mutant that has been earlier shown to be important for hepatocyte invasion and salivary gland infection in mosquitoes [14,25] showed only ~17% reduction in HepG2 cell binding in this study in comparison to wild type. The results of site-directed mutagenesis experiments in this paper thus suggested that amino acid residues other than those present in RII region play an important role in TRAP-HepG2 cell interaction. These mutants were also analysed for heparin binding, where no significant reduction in binding was observed. Together, these results suggest that PfTRAP binds to HepG2 cells using multiple modes; both heparin and non-heparin mediated interactions play roles in mediating TRAP interaction to HepG2 cells. Similar multiple interactions have been reported for a number of proteins involved in cell-cell attachment [26]. For example, extracellular matrix protein fibronectin simultaneously binds to heparin sulfate proteoglycans of syndecans and one or more integrins to induce cell spreading and focal adhesion formation [27]. Also a recent study showed that the extracellular domains of proteoglycans could bind to the protein ligands independent of heparin sulfate chains [28]. In the present study, an arginine residue (R281) in the RxRKR motif was also mutated, but the mutant protein failed to get secreted in the supernatant raising question about the misfolding of TRAP mutant molecule as suggested by Tossavainen and coworkers [29].

*In silico *analysis of TRAP from all the *Plasmodium *species revealed that TRAP molecules contain conserved PxxP motifs and many potential phosphorylation sites (Table 3). This suggests possible roles for TRAP in cell signaling once the sporozoite has entered liver cells. The phosphorylation status of PfTRAP and its subdomains was probed in presence of HepG2 cell extracts. TRAP A+RII and ECD are readily phosphorylated, while PfTRAP A-domain is not. The phosphorylation occurs at multiple sites that include serine, threonine and tyrosine residues. An interaction of PfTRAP was observed with Lyn, Lck and CrkL kinases, which are all able to phosphorylate TRAP. Taken together, TRAP binding, mutagenesis and phosphorylation data clearly suggest new roles for TRAP during sporozoite invasion of liver cells.

**Table 3:** PxxP sequence motifs in *Plasmodium *TRAP C-terminal regions

PfTRAP	RNI**PYSPLPP**; **PKHP**ER
PbTRAP	RDC**PPKP**; **PVIP**IK; **PDVP**VK; **PILP**IK; **PEIP**SK
PyTRAP	**PSNP**NK; RDC**PQIP**; **PVIP**NK; RRN**PNKP**; **PIIP**QK; KDE**PEIP**
PvTRAP	**PWDP**; **PLPP**
PkTRAP	**PVPPTVP**; **PENPENP**
PcTRAP	RNS**PMNP**

## Conclusion

Results presented in this study show that PfTRAP is a multifaceted molecule that plays important roles in sporozoite binding to HepG2 cells and possibly in signal transduction. At present, it is difficult to say whether the modification of PfTRAP occurs while it is still on the sporozoite surface (thereby supporting the sporozoite activation model of transmigration) [30-32] or once it has been shed along with heparan sulfate and localized into the cytoplasm of the host cell. However, these observations open new avenues for investigating the role of PfTRAP during the transmigration of sporozoite through the host cells or during the homing of sporozoites inside the HepG2 cells.

## Competing interests

The authors declare that they have no competing interests.

## Authors' contributions

PM and AS designed the experiments, RRA conducted them while all four wrote the paper.
